# Effects of online marketplace-sourced over-the-counter tooth whitening products on the colour, microhardness, and surface topography of enamel: an in vitro study

**DOI:** 10.1038/s41405-024-00253-0

**Published:** 2024-08-23

**Authors:** Radhika Agarwal, Nikki Vasani, Urmila Sachin Mense, Niharika Prasad, Aditya Shetty, Srikant Natarajan, Arindam Dutta, Manuel S. Thomas

**Affiliations:** 1 Manipal College of Dental Sciences Mangalore, Manipal Academy of Higher Education, Karnataka, Manipal 576104 India; 2 A.B.Shetty Memorial Institute of Dental Sciences (ABSMIDS), Nitte (Deemed to be University), Mangalore, 575018 India; 3 School of Dentistry, College of Biomedical and Lifesciences, Cardiff University, Cardiff, CF14 4XY UK

**Keywords:** Tooth whitening, Dental business

## Abstract

**Objectives:**

This study compared the whitening effect, microhardness, and enamel surface alterations of over-the-counter (OTC) tooth bleaching products with those of a dentist-prescribed at-home bleaching agent.

**Materials and methods:**

The products available on a popular online marketplace were comprehensively searched and then rated using a specific formula. The effects of the lowest-rated OTC agent (LRA) and the highest-rated OTC agent (HRA) were compared with those of a dentist-prescribed bleaching agent (DPA) on the enamel of extracted human teeth. The bleaching efficacy, post-bleaching microhardness changes, and morphological alterations were assessed by spectrophotometric analysis, Vickers hardness testing, and scanning electron microscopy (SEM) respectively. Statistical analyses included one-way ANOVA and post hoc tests, maintaining a significance level of *P* < 0.05.

**Results:**

The search of the online marketplace revealed 15 products. The LRA (Teeth Whitening Serum Gel, GEN, China) and the HRA (Bright White-Lovely Smile Premium Teeth Whitening strips, Ray of Smile, USA) were identified on the basis of their ranking. DPA resulted in significantly better tooth whitening than did LRA. The enamel microhardness was lower in the LRA treatment group (14.2%) than in the control and HRA treatment groups (8.84% and 7.26%, respectively). LRA also caused severe topographical alterations to the enamel.

**Conclusion:**

Compared with the poorly rated product, the dentist-prescribed tooth bleaching product resulted in greater colour improvement, less microhardness reduction, and surface changes. The highest-rated product was comparable with the dentist-prescribed agent in this study.

## Introduction

Dental patients seek care for several reasons, such as pain, swelling, improved function, and the appearance of teeth. The desire to enhance dental aesthetics is important because it is related to the psychosocial status of dental patients [1]. In the maxillary anterior segment, the colour of the teeth is a key factor that determines the cosmetic value of a patient’s facial appearance. Patient requests for improvement of tooth colour are frequently addressed conservatively by the provision of in-office or home bleaching techniques prescribed by the dental practitioner, which also help provide long-lasting improvements to the user’s quality of life [2].

Accessing dental care is often difficult in different parts of the world [3–5] and can be influenced by financial constraints [6, 7]. While professional tooth whitening can lead to a desirable improvement in tooth colour, this method is frequently provided privately and can be expensive [8]. Over-the-counter (OTC) whitening products have therefore been developed and are considered low-cost alternatives that do not require the supervision of a dentist [9–11].

All such whitening products (either professionally applied, dentist-prescribed, or available OTC) include chemicals that can potentially disintegrate chromogens, which leads to tooth colour improvement. However, such chemicals can also adversely affect the mechanical and physical characteristics of hard dental tissues [12]. Owing to their ease of purchase and lower cost, the use of OTC bleaching agents in the form of gels, paints, pencils, and strips has become popular. These products have been shown to have side effects such as mucosal and tooth sensitivity [13]. Moreover, as OTC products are used without professional oversight, the potential for misuse with adverse clinical consequences is a cause for concern [14]. In addition to drugstores and supermarkets, OTC tooth whitening agents are available online through various e-commerce websites. The information available online for consumers may not be accurate or adequate. Therefore, unsupervised use of these products can cause more harm than good [15].

Hence, this study compared the bleaching efficiency, enamel microhardness, and enamel surface alterations of OTC tooth bleaching products available online with those of a dentist-prescribed at-home bleaching agent (DPA). The pH of the bleaching agents used in the study was also determined.

## Materials and methods

### Study approval and design

This in vitro study commenced after ethical approval was obtained from the Institutional Ethics Committee (IEC reference number: *22091*).

### Sample size

According to the key article by Al-Angari et al. [16], the expected standard deviation was 3.7. With an alpha error of 1%, a power of 95%, and maintaining an effective difference to demonstrate a clinically significant difference of 5, the number of samples required in each group was calculated to be ten using PASS 11.0.7 (Kaysville, Utah, USA).

### Selection of the experimental bleaching agents

**Fig. 1:**
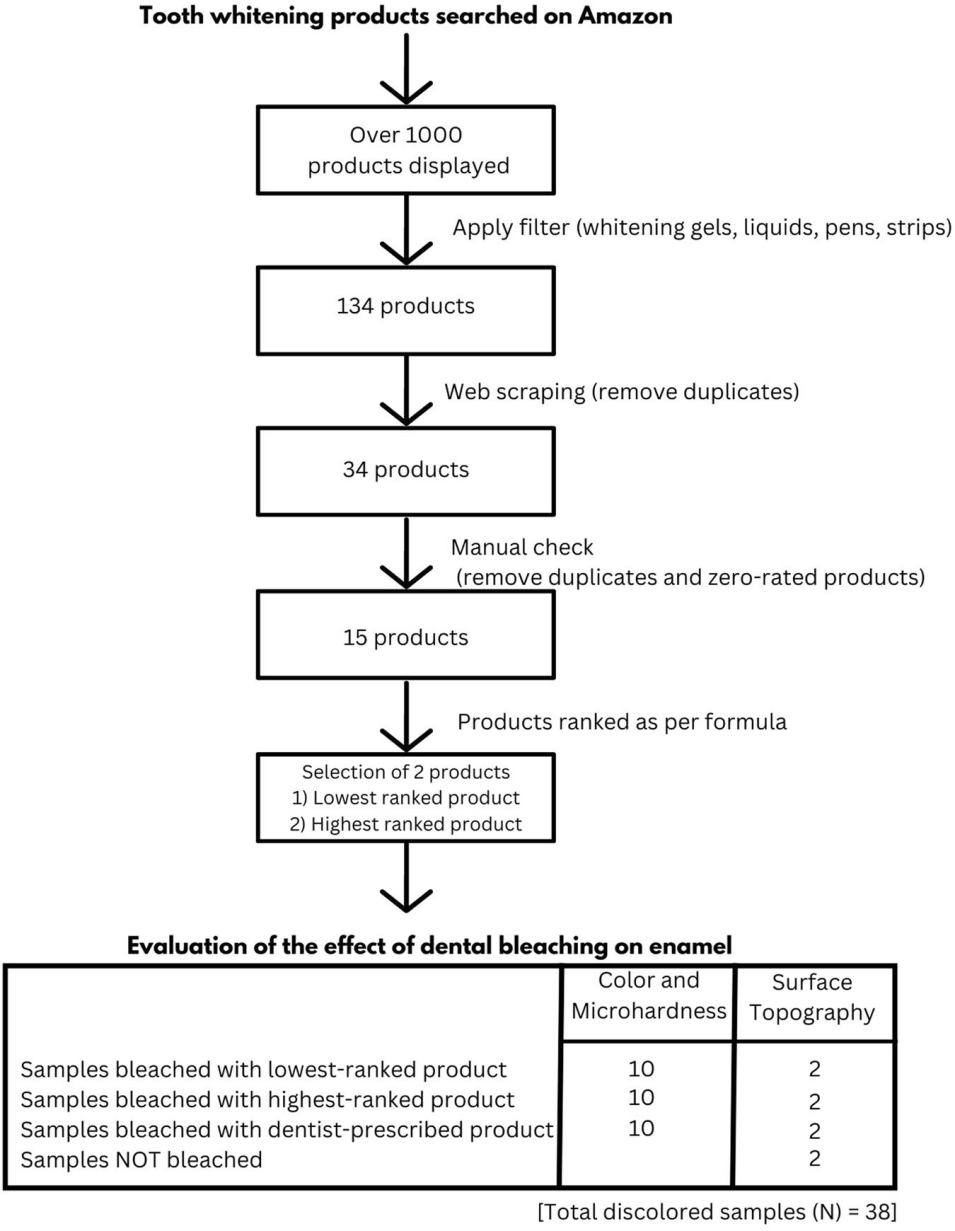
Flowchart illustrating the selection of bleaching agents from the online marketplace and the evaluation of the effects of selected products on dental enamel.

A comprehensive internet search was conducted to identify teeth whitening products available on a popular online marketplace, Amazon.com [17]. Only tooth-whitening products applied to teeth that do not require mechanical action, such as whitening gels, liquids, pens, and strips, were included in the study. Toothpastes, powders, or foams that require mechanical activation, such as brushing, were excluded. The search was independently performed on June 25, 2023, by two investigators (R.A. and N.V.), and any discrepancies were resolved through discussion (Fig. 1).

A web-scraping tool accessed from the website www.webautomation.io was used (by N.V.) to conduct an extensive data collection process, which included resolving duplicate and kit entries [18]. Even after web scraping and deduplication, it is common to find duplicates because of dynamic content loading, inconsistent URLs, and scraper logic errors. Additionally, websites can have overlapping data across pages, leading to residual duplicates. Consequently, a few duplicates had to be removed manually even after the web scraping process (by R.A.). In the case of duplicate products with varying reviews, the review with the highest rating was selected. Additional information on the product form, primary ingredients, ratings, usage instructions, and approval stamps was manually added by the two investigators (N.V., R. A.) (Table 1). Bleaching agents with no reviews were eliminated.

**Table 1:** Selected list of over-the-counter teeth whitening products from an online marketplace.

No.	Description	Form	No. of ratings	Amazon rating out of 5	Rating from the formula	Primary ingredient	Instruction of use
1.	Bright White- Lovely Smile Premium Teeth Whitening strips (Ray of Smile, USA)	Strip	2963	4.3	2.06	Hydrogen Peroxide, Menthol, Hydroxypropyl cellulose, PVP, Glycerol, Alcohol, Water	Absent
2.	Oral Essentials Teeth Whitening Mouthwash (Oral Essentials, USA)	Liquid	3317	4.2	1.97	Coconut oil, Lemon, Sage oil, Lemon peel oil, Dead Sea salt	Absent
3.	Perfora Teeth Whitening Pen Peroxide Free (Onuge Personal Care, China)	Gel	357	4.1	1.84	PAP, Nano Hydroxyapatite, Alovera, Honeysuckle Extract, Sodium bicarbonate	Present
4.	Agaro Dazzle Instant Teeth Whitening Strips (Universal Corporation Ltd., China)	Strip	14	3.9	1.78	Mint, other details N.A.	Present
5.	Daybreak Teeth Whitening Strips (Nanchang Dental Bright Co. Ltd, China)	Strip	43	3.8	1.67	PAP, Hydroxyapatite	Present
6.	Daybreak Teeth Whitening Pen (Nanchang Dental Bright Co. Ltd, China)	Gel	46	3.7	1.64	PAP, Hydroxyapatite	Present
7.	Techworld Portable Advanced Professional Teeth Whitening Strips (Techworld, China)	Strip	13	3.6	1.64	Triethanolamine, Glycerine, Carbomer, Peppermint Oil	Present
8.	Teeth-a-bit Teeth Whitening Snow White Gel (Trillion-Bits India Pvt., Ltd., India)	Gel	29	3.7	1.5	Papain (Carica Papaya), Bromelain (Ananas comosus), Menthol Carbopol 981, Glycerine, Liquid sorbitol, Triethanolamine, Propylene Glycol	Present
9.	Lanbena Teeth Whitening Essence Serum	Liquid	133	3.4	1.38	Hydrated Silica, other details N.A.	Present
10.	Protouch Pearl White Teeth Whitening Pen (Protouch, India)	Gel	795	3.3	1.3	Citrus lemon peel extract, Turmeric root extract, Spearmint leaf oil, other details N.A.	Present
11.	SmiloShine Teeth Whitening Charcoal Gel (Novateor Research Laboratories Pvt. Ltd., India)	Gel	31	3.3	1.27	Activated charcoal, Menthol	Present
12.	Yeesport teeth whitening strip (Yeesport, China)	Strip	134	3.1	1.23	PVP, Glycerine, Water, Polysorbate-80, Flavour Citric Acid, Maltodextrin, Cellacefate	Present
13.	Bonayu Teeth Whitening Strips (Bonayu Lifesciences Ltd., India)	Strip	78	2.9	1.16	Activated Charcoal, Coconut oil, Maltodextrin, Hydroxypropyl cellulose, Micro crystalline cellulose, Sorbitol, Ste viol Glycoside, Menthol	Present
14.	Park Daniel Teeth Lightening Foam (Satyam Enterprises, India)	Mousse	78	2.4	0.69	Mint Extract, Extracts of Water, Glycerol, Citrus, Junos extract, Xylitol, Baking Soda, Essence.	Present
15.	Teeth Whitening Serum Gel (GEN, China)	Liquid	51	2.3	0.67	Teeth Whitening Serum, other details N.A. (^a^Aqua, Acetum, Butylene glycol, PEG-40 Hydrogenated castor oil, Hydroxyethyl cellulose, Phenoxyethanol, Menthol, Disodium phosphate, Polysorbate 60, Sodium phosphate, Ethylhexylglycerin).	Present

*N.A.* not available, *PVP* polyvinylpyrrolidone, *PAP* phthalimidoperoxycaproic acid.

^a^Composition as listed on the product packaging.

Finally, each selected product was ranked using the formula below, which utilized customer evaluations and ratings [19].

$$Ratin{g}_{x}\frac{{\sum }_{i=1}^{N}\,\{Review\,Ratin{g}_{i}\times (1+ThumbU{p}_{i})\}}{N+{\sum }_{i=1}^{N}\,ThumbU{p}_{i}}$$

(*N* is the total number of reviews for product x, *Review Rating* denotes the overall assessment given for the product on Amazon, and *ThumbUp* represents the number of ratings falling within the three- to five-star range).

The tooth bleaching agent with the lowest rating (LRA) and the highest rating (HRA) was selected for this study.

### Sample selection and storage

Freshly extracted, intact permanent maxillary anterior human teeth that did not have any visible cracks, staining, decay, demineralization, enamel imperfections, fluorosis, hypoplastic defects, or dental restorations were included in the study. Thirty-eight such teeth were collected and stored at room temperature in a distilled water solution before the testing phase.

### Sample preparation

The roots of the teeth were sectioned 2 mm apical to the cemento-enamel junction using a low-speed diamond disc (Frank Dental GmbH, Germany) under water cooling. The coronal portion of the tooth was embedded in acrylic, with the buccal surface facing outwards. A 5 mm circular area on the buccal enamel was prepared by grinding with 400-grit silicon carbide abrasive paper followed by 600 and 1200-grit aluminium oxide paper for polishing.

### Staining procedure

All thirty-eight samples were artificially stained with a coffee extract solution prepared by dissolving 5.5 g of instant coffee powder (Bru Instant Coffee Powder, India) in 250 ml of boiling water at 100 °C [20]. The samples were immersed in the solution for three days at 37 °C, after which the solution was replaced daily. The samples were subsequently washed and ultrasonicated in distilled water to remove superficial stains to achieve shades matching A3 or higher, as per the Vita Classical Shade Guide arranged in a value-based format.

### Grouping

Among the thirty-eight samples, 36 were randomly divided into three different tooth whitening groups. The groups were as follows (Fig. 1):

Group I: Experimental group–The LRA (dispensed as a solution) was applied to the tooth surface using a cotton swab and left undisturbed for twenty minutes, as per the manufacturer’s instructions. This process was repeated three times over one week.

Group II: Experimental group–The HRA (dispensed as a bleaching strip) was applied to the tooth surface twice daily and left in place for 30 min at a time. This process was repeated daily over one week, as specified by the manufacturer.

Group III: Positive control group – DPA (20% carbamide peroxide (CP), Opalescence, Ultradent, USA) was used as per the manufacturer’s instructions and was applied to the tooth surface at a thickness of 1–2 mm for four hours every day. This process was repeated daily throughout the week.

After each bleaching cycle, the samples were thoroughly rinsed and then stored in distilled water in an incubator at 37 °C [21, 22].

### Colour measurement

The tooth shade analysis involved recording the shade of thirty anterior teeth (10 in each group) using a handheld digital spectrophotometer (VITA Easyshade V, VITA Zahnfabrik H. Rauter GmbH & Co. KG, Germany) in VITA classical A1–D4 shade mode. The shade of each tooth was represented as a shade guide unit (SGU) on the basis of the value-based arrangement of the VITA Classical scale. Each of the 16 tabs of the shade guide was assigned a number from 1 to 16, arranged from the highest value (B1 having an SGU of 1) to the lowest value (C4 having an SGU of 16) [23]. Although this scale is not strictly linear, it was considered continuous and approximately linear for the purpose of analysis.

The shade of each sample was evaluated before bleaching and one day post-bleaching at a consistent location on the middle third of the labial surface, following the guidelines of the American Dental Association (ADA) [24]. Each VITA classical shade obtained was converted to an SGU. The change in colour after bleaching was calculated as the difference in the number of SGUs (ΔSGU) from the lighter end of the value-oriented list of shade tabs [25].

### Microhardness measurements

Microhardness measurements were conducted both before the application of the bleaching agent and 24 h after the completion of the bleaching regimen for each of the samples. The Vickers hardness number (VHN) for each sample was evaluated via a surface microhardness tester (Shimadzu HMV-2000, Germany). For each sample, Vickers hardness measurements were taken at three different locations on the surface, with a 25-g load applied for 20 s at each location. The average of three measurements represented the VHN for each sample [26].

### Scanning electron microscopy (SEM)

The enamel surface topography was assessed using SEM (Carl Zeiss Model Number – EVO18 Special Edition, Carl Zeiss, Cambridge, UK) after sputter coating the samples. Two teeth that were not bleached were used as controls. Two teeth from each group (LRA, HRA, and DPA) that had been bleached for 24 h but whose microhardness was not tested were evaluated for micromorphological alterations.

### pH evaluation

The pH of the bleaching agents was assessed using a digital pH metre (Manti Lab Solutions Panchkula, India). Each bleaching agent was diluted 1:2 with distilled water to ensure uniformity before pH measurement. As the HRA was available as a strip, the bleaching agent was scraped off the strip before dilution. The pH of the distilled water was also measured before dilution. pH measurements were conducted in triplicate for each bleaching agent, and the average pH was recorded.

### Statistical analysis

The data were tabulated and analysed via statistical software (SPSS Version 20, IL, USA). Intergroup comparisons of shade and microhardness were assessed through one-way ANOVA with subsequent Tukey’s HSD post hoc test. Intragroup comparisons of pre- and post-bleaching shade and microhardness were evaluated using paired *t-tests*. A statistical significance level of *P* < 0.05 was applied.

## Results

### E-portal bleaching products

A comprehensive search of the Amazon e-portal for teeth whitening products was performed, resulting in a list of more than 1000 products. After web scraping and selection on the basis of the inclusion and exclusion criteria, the number of products was reduced to 15, with Amazon ratings ranging from 2.3 to 4.3 out of 5. (Fig. 1 and Table 1)

Among the 15 products, 13 were accompanied by comprehensive usage instructions, ensuring clarity in their application. On the basis of the criteria and formula outlined in the methodology section, the best and worst products were identified. The HRA (rating = 2.06) was available as a strip (Bright White-Lovely Smile Premium Teeth Whitening strips (Ray of Smile, USA), and the LRA (rating = 0.67) was supplied as a liquid [Teeth Whitening Serum Gel, GEN, China] (Table 1).

### Shade alterations

According to the analysis of pre-bleaching and post-bleaching shade changes, significant changes were observed among the three experimental groups (Table 2). The greatest change in shade (ΔSGU) was detected in the DPA group, indicating the most noticeable shift in shade post-bleaching. Conversely, the group with the least observed shade alteration was the LRA, indicating a relatively minimal colour change. A statistically significant difference was detected between LRA (3.8 ± 3.12) and DPA (8.9 ± 3.64) (*P* < 0.05) (Table 3).

**Table 2:** Intragroup comparisons of shade and microhardness.

Groups	Evaluation criteria	*N*	Mean ± SD	*t-*test	*P* value
Shade evaluation^a^					
Group I (LRA)	Pre-bleaching shade	10	9.6 ± 3.98	3.85	**0.004**
	Post-bleaching shade	10	5.8 ± 3.29		
Group II (HRA)	Pre-bleaching shade	10	10.8 ± 3.05	6.16	**<0.001**
	Post-bleaching shade	10	3.9 ± 1.2		
Group III (DPA)	Pre-bleaching shade	10	12.4 ± 2.22	7.74	**<0.001**
	Post-bleaching shade	10	3.5 ± 1.96		
Microhardness evaluation^b^					
Group I (LRA)	Pre-bleaching microhardness	10	209.33 ± 39.8	4.64	**0.001**
	Post-bleaching microhardness	10	179.71 ± 42.01		
Group II (HRA)	Pre-bleaching microhardness	10	223.49 ± 48.67	1.28	0.234
	Post-bleaching microhardness	10	195.94 ± 39.87		
Group III (DPA)	Pre-bleaching microhardness	10	183.97 ± 28.37	3.06	**0.014**
	Post-bleaching microhardness	10	167.09 ± 26.65		

^a^Mean values in the shaded guide unit (SGU).

^b^Mean values of the Vickers hardness number (VHN).

Bold signifies statically significant difference (*P* value < 0.05).

**Table 3:** Changes in shade between different bleaching agents [measured in shaded guide units (SGUs)].

Groups	Group I: LRA (*N* = 10) Mean ± SD	Group II: HRA (*N* = 10) Mean ± SD	Group III: DPA (*N* = 10) Mean ± SD	*P* value
Pre-bleaching shade	9.6 ± 3.98	10.8 ± 3.05	12.4 ± 2.22	0.159
Post-bleaching shade	5.8 ± 3.29	3.9 ± 1.2	3.5 ± 1.96	0.202
ΔSGU^*^	3.8 ± 3.12^a^	6.9 ± 3.54^a,b^	8.9 ± 3.64^b^	**0.009**

^*^If two values share a letter, they are not significantly different. ΔSGU between Group I and Group III has a significant difference (*P* value = 0.007).

Bold signifies statically significant difference (*P* value < 0.05).

### Alterations in the microhardness

A significant reduction in microhardness was found after the completion of bleaching with LRA and DPA (*P* < 0.05) but not with HRA (*P* > 0.05) compared with the microhardness recorded before bleaching within each group (Table 2).

The overall reduction in microhardness was most pronounced with LRA, and the smallest difference occurred with DPA. However, these changes were not statistically significant (*P* > 0.05) (Table 4).

**Table 4:** Changes in the microhardness [based on the Vickers hardness number (VHN)] between bleaching agents.

Hardness evaluation	Group I: LRA (*N* = 10) Mean ± SD	Group II: HRA (*N* = 10) Mean ± SD	Group III: DPA (*N* = 10) Mean ± SD	*P* value
Pre-bleaching microhardness	209.33 ± 39.8	223.49 ± 48.67	183.97 ± 28.37	0.099
Post-bleaching microhardness	179.71 ± 42.01	195.94 ± 39.87	167.09 ± 26.65	0.232
Overall difference	29.62 ± 20.18	27.55 ± 68.23	16.88 ± 17.46	0.35
Percent difference (%)	14.2 ± 9.16	7.26 ± 30.83	8.84 ± 8.65	0.407

*N* sample size.

### Alterations in enamel surface morphology

**Fig. 2:**
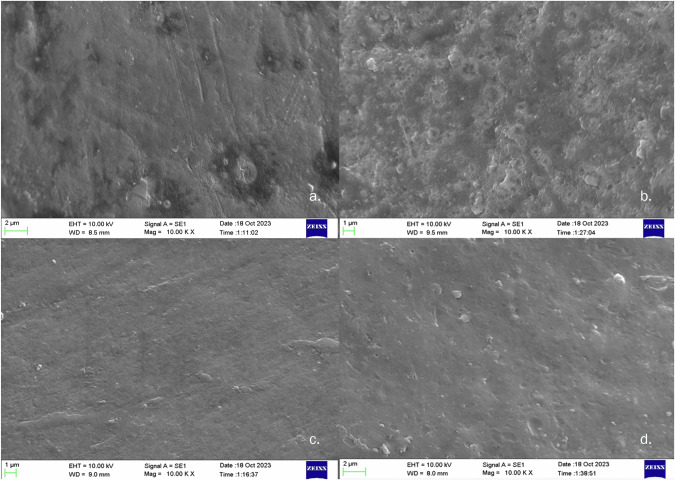
Scanning electron microscopy (SEM) images at ×10^3^ magnification. **a** SEM image of a sample before bleaching, **b** SEM image of a sample bleached with the LRA, **c** SEM image of a sample bleached with the HRA, and **d** SEM image of a post-bleaching sample from the DPA group.

SEM evaluation of the enamel surfaces revealed morphological changes in all the samples after bleaching (Fig. 2a–d). SEM analysis of the pre-bleached samples (magnification = 10^3^×) revealed relatively smooth surfaces with a few dark spots (Fig. 2a). The LRA bleached surfaces presented severe pitting on the enamel surface (Fig. 2b), whereas the HRA bleached tooth presented a smooth enamel surface (Fig. 2c). In the control group, where 20% CP was utilized (DPA), only mild pitting and minor surface irregularities were observed on the enamel surface (Fig. 2d).

### pH

The pH of the bleaching agents was evaluated using a digital pH metre, revealing noticeable differences among all three products. In the control group, after triplicate measurements, the average pH was 7.21, whereas for the HRA and the LRA, it was 7.44 and 4.11, respectively.

## Discussion

OTC products available through online marketplaces offer consumers convenient and affordable oral healthcare options without a prescription. However, these platforms often struggle to verify the authenticity and quality of products sold by third-party sellers. Additionally, incomplete product descriptions can lead to consumer misunderstanding or even misuse [27, 28]. Owing to the lack of research on bleaching agents sold online, this study aimed to gather information on these products from a leading online marketplace and assess their bleaching effectiveness, as well as their impact on enamel microhardness and surface topography, compared with a dentist-prescribed bleaching agent.

The decision to focus the study on tooth whitening products available in Amazon, India, was planned because of its considerable reach and influence in the Indian market. Amazon, India, stands out for its expansive product inventory, competitive pricing, and user-friendly shopping interface [29]. The platform enables customers to compare products thoroughly and access and assess customer feedback and reviews [30]. Additionally, Amazon uses artificial intelligence to summarize customer reviews of a product as a summary paragraph, which helps present data succinctly. Hence, the popularity of this online marketplace amongst shoppers made it the ideal choice for the current investigation.

Comprehensive data collection for tooth whitening products on Amazon was conducted via a multistep approach that included Web data scraping, a fundamental tool for extracting online content [31]. The assessment of the information on the website revealed that many products lacked detailed information regarding formulation, safety issues, and adequate usage instructions. This lack of information is concerning, as it can affect the safety and reliability of these products.

The ratings for these products were derived from a formula by Sarkar and Ahmad to identify the best and worst products [19]. The worst-rated product was a liquid, whereas the best-rated product was a strip. The control group was treated with a 20% carbamide peroxide solution, which is commonly prescribed by dental professionals [32, 33]. The enamel samples were randomly assigned to the three groups and stored in distilled water to prevent dehydration before and after the bleaching process. Artificial saliva was avoided to prevent interference with the bleaching agent’s effects [21, 22]. To ensure accurate results, the samples were stored in deionized water.

Consumer choices are most frequently based on the achievement of intended results, which would be tooth shade improvement for the products investigated in the current study. Shade parameters, including shade guide units (SGUs) and shade change (ΔSGU), were recorded using the VITA Easyshade V, a device known for its consistent and precise colour measurement capabilities [34, 35]. SGU change was considered instead of overall colour change (Delta E) with the Commission Internationale de l'Éclairage Lab (CIElab) system because it directly corresponds to the shade guides commonly used by dentists [36]. Significant changes in pre-bleaching and post-bleaching shades were observed in the three groups. Several factors may influence improvements in shade, including the main active ingredient of the tooth whitening product. Hydrogen peroxide and carbamide peroxide are recognized as well-known bleaching agents. The maximum shade change (ΔSGU) in the current study was identified in the control group (DPA) because of the potent oxidizing properties of carbamide peroxide, which effectively breaks down discolouration-causing molecules [37, 38]. Conversely, LRA, the worst-rated product, presented a minimal ΔSGU value, likely due to the absence of peroxides in its composition. Significant differences were found between LRA and DPA in the intergroup comparisons. The active ingredient present in the LRA is Acetum, which is commonly known as vinegar. Vinegar has been used as a home remedy to obtain whiter teeth [39]. However, the shade change resulting from its use is not predictable, as the whitening caused is due to the dimerization action of the acidic ingredient [40].

HRA also effectively caused shade changes, which could be attributable to the presence of hydrogen peroxide as an active ingredient in the product, but its exact concentration in the strip was not determined online. Generally, bleaching strips have a variable concentration of hydrogen peroxide (5–15%) [41]. A comparison of the improvement in shade between the 20% carbamide peroxide gel and the 6% hydrogen peroxide strips revealed better efficacy for the former agent after one week, although the strips were also able to improve shade [42]. The overall hydrogen peroxide concentrations were nearly equivalent in both products; therefore, the dispensing method and duration of application might have influenced improvements in shade. Other studies, supported by moderate evidence, also concluded that the differences in colour change between dentist-supervised at-home bleaching techniques and over-the-counter whitening strips were minimal [43]. The better whitening efficiency of the bleaching strip (HRA) than the paint-on solution (LRA) in the present study could be attributed to the longer contact time and the presence of hydrogen peroxide in the strip. Previous studies comparing paint-on gels with whitening strips also revealed that the strips had better results because of intimate contact with teeth and longer application times [44, 45]. Nonetheless, because the indiscriminate use of whitening strips can lead to tooth sensitivity and gingival irritation, clinical examination of the dental and periodontal conditions is important before the use of strips [41, 43].

The VHN of each sample was evaluated using a surface microhardness tester, which indirectly reflects the mineral content and tissue integrity of the enamel [46]. Factors such as the concentration, application time, and formulation of the bleaching agent can affect the microhardness of dental hard tissues [47–49]. The most noticeable overall reduction in microhardness was observed when the enamel was bleached with the worst-rated agent. This could be because of enamel demineralization caused by the product’s low pH owing to the presence of acetic acid. A previous study has also reported a maximum reduction in microhardness after bleaching with a non-peroxide bleaching agent, where one of the constituents was an acidic agent, namely citric acid [50]. Conversely, the lowest percentage difference in microhardness was reported with HRA, which was not significantly different from DPA. The post-bleaching microhardness can be influenced by the storage medium [51]. In the present investigation, where samples were stored in distilled water, the reduction in microhardness reported in the present study may be compensated for clinically by salivary minerals. Further research in this field is therefore warranted.

Topographical changes in the enamel were observed through scanning electron microscopy (SEM), a common method for assessing the effects of bleaching agents on hard dental tissue surfaces [52, 53]. A qualitative evaluation of the SEM image (Fig. 2a) of a prebleached sample revealed smooth surfaces with few dark spots and uneven areas, which were likely due to debris accumulation during polishing. With the use of DPA, which contains 20% carbamide peroxide, the post-bleaching enamel surface (Fig. 2d) showed intermittent depressions and mild surface irregularities after bleaching. These findings are similar to those of other studies that have shown that 20% carbamide peroxide did not significantly alter the enamel microstructural surface, indicating minimal ultrastructural changes [54, 55].

However, the sample bleached with the LRA (Fig. 2b) exhibited severe surface pitting. This can be explained by the low pH of the product, thus accounting for the pitted surface, which is very similar to an etched enamel pattern. The HRA resulted in a smooth enamel surface (Fig. 2c), which could be attributed to its shorter application time and nearly neutral pH. These findings are similar to those of the DPA in the present study and are also concordant with other SEM studies that evaluated both dentist-prescribed and OTC tooth whitening agents supplied in different forms, such as toothpastes, mouthwashes, and pens [47, 49]. The surface characteristics could be influenced by the overall duration of use of the bleaching agent. In the present study, bleaching was performed over one week only, which had minimal influence on the enamel surface. However, patients may bleach teeth longer than a few weeks, which could adversely influence the smoothness of the enamel surface [56]. These findings regarding surface changes are significant since increased surface irregularities may capture dietary chromogens from the oral environment and potentially lead to worsening tooth colour. Further studies that correlate postbleaching surface roughness with dietary chromogens are therefore needed.

This study is constrained by several factors. First, it relies solely on one online marketplace for selecting products, potentially limiting the wider applicability of the findings. Additionally, incomplete data on the composition of different products may have compromised the precision of result interpretation. Moreover, as an in vitro study, it did not account for the dynamic oral environment, such as the impact of saliva, the oral microflora, and temperature on the effects of bleaching agents on the tooth substrate. To address these limitations, in future research, studies should involve multiple online marketplaces to enhance generalizability and encompass a broader range of products. It is also crucial to obtain comprehensive and detailed information on product compositions to ensure a more accurate interpretation of results. Furthermore, researchers should assess regulatory standards for whitening products across different global regions to gauge their effectiveness in ensuring patient safety and efficacy. These steps can help advance understanding and application in the field of dental care.

## Conclusions

The current investigation showed that the dentist-prescribed bleaching agent had near neutral pH and showed better shade improvement, lesser microhardness reduction, and minimal enamel surface change as compared with a poorly rated product which was sourced from an online marketplace. The highly rated online product, available as a bleaching strip, had results that were similar to the dentist-prescribed agent. These differences could be attributed to the composition of the product, pH, method and duration of application.

## Data Availability

The datasets used and/or analysed during the current study are available from the corresponding author upon reasonable request.
